# Supplementary material for: FosL1 Is a Novel Target of Levetiracetam for Suppressing the Microglial Inflammatory Reaction
Source: Int J Mol Sci. 2021 Oct 11;22(20):10962. doi: 10.3390/ijms222010962 (PMC8537483; doi:10.3390/ijms222010962)
Supplement: Supplementary file 1 [file ijms-22-10962-s001.zip › ijms-1380973-supplementary.pdf]

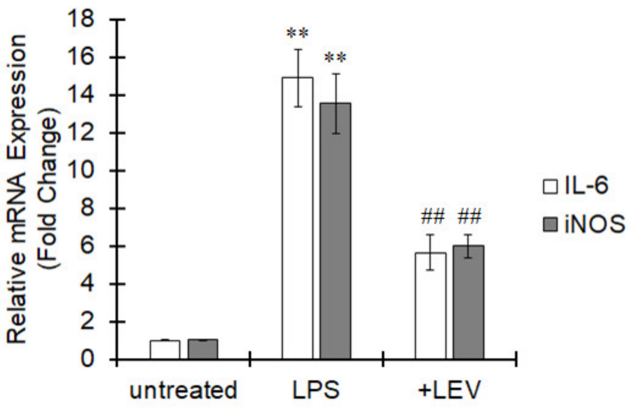

Figure S1. Suppression of LPS-induced expression of IL-6 and iNOS by treatment with LEV in BV-2 cells. BV-2 cells were pretreated with 10 mM LEV for 20 min, and subsequently 10 ng/mL LPS was added for 24 h. Total RNA was extracted, and the mRNA expression of IL-6 and iNOS was evaluated by real-time PCR. Primer sequences were listed as Supplementary Table S1. The values are presented as the mean ± S.E. of 5 separate experiments. The data were analyzed using ANOVA followed by Student's t-test. ** $p < 0.01$ vs. the untreated group and ## $p < 0.01$ vs. the LPS-treated group.

Table S1. Primers for IL-6 and iNOS detection.

Target	Forward Primer	Reverse Primer
Mouse IL-6	TCTCTGCAAGAGACTTCCATCC	TTGTGAAGTAGGGAAGGCCG
Mouse iNOS	TCCTGGACATTACGACCCCT	CTCTGAGGGCTGACACAAGG

Table S2. Motifs found in JASPAR database.

Name	Sequence	Action
DREME_001	AGTCATC	Increased by LPS
DREME_016	AAATCACTCAC	Increased by LPS
AMD_001	ATGACTGTGAGTGATTTTCTGAG	Increased by LPS
AMD_003	GTCTGAGGCAGGGGGATGACTG T	Decreased by LEV
